# Subcellular Location of Tirapazamine Reduction Dramatically Affects Aerobic but Not Anoxic Cytotoxicity

**DOI:** 10.3390/molecules25214888

**Published:** 2020-10-22

**Authors:** Chris P. Guise, Maria R. Abbattista, Robert F. Anderson, Dan Li, Rana Taghipouran, Angela Tsai, Su Jung Lee, Jeff B. Smaill, William A. Denny, Michael P. Hay, William R. Wilson, Kevin O. Hicks, Adam V. Patterson

**Affiliations:** 1 Auckland Cancer Society Research Centre, School of Medical Sciences, The University of Auckland, Private Bag 92019, Auckland 1142, New Zealand; chrispguise@hotmail.com (C.P.G.); m.abbattista60@gmail.com (M.R.A.); r.anderson@auckland.ac.nz (R.F.A.); leoyns@icloud.com (D.L.); rana6586@hotmail.com (R.T.); a.tsai@auckland.ac.nz (A.T.); l.sujung@gmail.com (S.J.L.); j.smaill@auckland.ac.nz (J.B.S.); b.denny@auckland.ac.nz (W.A.D.); m.hay@auckland.ac.nz (M.P.H.); wr.wilson@auckland.ac.nz (W.R.W.); k.hicks@auckland.ac.nz (K.O.H.); 2 Maurice Wilkins Centre for Molecular Biodiscovery, The University of Auckland, Private Bag 92019, Auckland 1142, New Zealand

**Keywords:** tirapazamine, DNA-targeted cytotoxin, hypoxia-activated prodrug, cytochrome P450 oxidoreductase, cell nucleus, endoplasmic reticulum, cell membrane, DNA damage-response

## Abstract

Hypoxia is an adverse prognostic feature of solid cancers that may be overcome with hypoxia-activated prodrugs (HAPs). Tirapazamine (TPZ) is a HAP which has undergone extensive clinical evaluation in this context and stimulated development of optimized analogues. However the subcellular localization of the oxidoreductases responsible for mediating TPZ-dependent DNA damage remains unclear. Some studies conclude only nuclear-localized oxidoreductases can give rise to radical-mediated DNA damage and thus cytotoxicity, whereas others identify a broader role for endoplasmic reticulum and cytosolic oxidoreductases, indicating the subcellular location of TPZ radical formation is not a critical requirement for DNA damage. To explore this question in intact cells we engineered MDA-231 breast cancer cells to express the TPZ reductase human NADPH: cytochrome P450 oxidoreductase (POR) harboring various subcellular localization sequences to guide this flavoenzyme to the nucleus, endoplasmic reticulum, cytosol or inner surface of the plasma membrane. We show that all POR variants are functional, with differences in rates of metabolism reflecting enzyme expression levels rather than intracellular TPZ concentration gradients. Under anoxic conditions, POR expression in all subcellular compartments increased the sensitivity of the cells to TPZ, but with a fall in cytotoxicity per unit of metabolism (termed ‘metabolic efficiency’) when POR is expressed further from the nucleus. However, under aerobic conditions a much larger increase in cytotoxicity was observed when POR was directed to the nucleus, indicating very high metabolic efficiency. Consequently, nuclear metabolism results in collapse of hypoxic selectivity of TPZ, which was further magnified to the point of reversing O_2_ dependence (oxic > hypoxic sensitivity) by employing a DNA-affinic TPZ analogue. This aerobic hypersensitivity phenotype was partially rescued by cellular copper depletion, suggesting the possible involvement of Fenton-like chemistry in generating short-range effects mediated by the hydroxyl radical. In addition, the data suggest that under aerobic conditions reoxidation strictly limits the TPZ radical diffusion range resulting in site-specific cytotoxicity. Collectively these novel findings challenge the purported role of intra-nuclear reductases in orchestrating the hypoxia selectivity of TPZ.

## 1. Introduction

Hypoxia is a common pathophysiological feature of solid tumors that plays a central role in cancer progression and resistance to therapy [1,2,3,4]. Considerable efforts have gone into the development of effective therapies to target tumor hypoxia, such as exposing patients to hyperbaric oxygen [5,6], the use of 2-nitroimidazole radiosensitizing agents [7] or the development of hypoxia-activated prodrugs (HAP) to selectively target hypoxic tumor cells [8,9]. One of the most well studied examples of a HAP is the aromatic di-*N*-oxide tirapazamine (TPZ), which can undergo metabolic reduction in hypoxic cells to generate cytotoxic DNA damaging radical species [10,11,12,13,14,15].

TPZ has been extensively evaluated in the clinic; encouraging results were obtained from phase II clinical trials in non-small cell lung cancer (NSCLC) [16] and advanced head and neck squamous cell carcinoma (HNSCC) [17]. A sub-study of the head and neck trial utilized FMISO PET imaging to identify patients with high levels of tumor hypoxia and showed these patients benefited most from treatment with TPZ [18]. However, a subsequent Phase III trial in HNSCC showed no evidence for improved survival or local control upon addition of TPZ to chemo-radiotherapy [19]. A lack of efficacy at Phase III was likely due to a number of factors, including an absence of hypoxia pre-screening to target appropriate patient groups [18], challenges with radiotherapy protocol compliance [20], poor tumor penetration of TPZ [21] and the impact of HPV infection on sensitivity to TPZ [22]. Consideration of these factors will lead to improvements in trial design and will therefore assist in the clinical development of TPZ analogues that exhibit superior diffusion properties [23]. The clinical potential of this class of HAP warrants elucidation of the precise role of cellular enzymes in their metabolic reduction.

**Figure 1:**
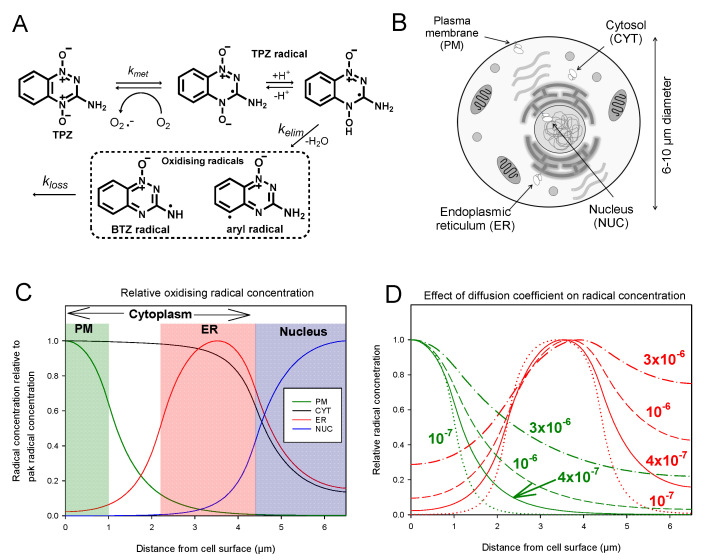
Reaction-diffusion modelling predicts non-isotropic distribution of cytotoxic TPZ radicals following TPZ activation under anoxia in sub-cellular compartments: (**A**) Scheme showing the metabolic activation of TPZ, which undergoes one-electron reduction to generate the oxygen-sensitive radical anion. The protonated radical eliminates water to generate reactive oxidizing benzotriazinyl (BTZ) and aryl radicals, in the absence of oxygen; (**B**) Schematic representation showing sources of one-electron TPZ reduction explored in the present study; (**C**) Reaction-diffusion modelling of the intracellular steady state TPZ-derived oxidizing radicals (relative to peak concentration) when generated in different subcellular compartments (PM green, ER red, CYT black, NUC blue), assuming an intracellular diffusion coefficient, *D*, of 4 × 10^−7^ cm^2^ s^−1^ for TPZ and its radicals; (**D**) Model results for *D* = 10^−7^ cm^2^ s^−1^ (dotted line), 4 × 10^−7^ cm^2^ s^−1^ (solid line), 10^−6^ cm^2^ s^−1^ (dashed line) or, 3 × 10^−6^ cm^2^ s^−1^ (dot-dash line) for activation at the PM (green) or ER (red).

Reduction of TPZ can be mediated by both one- and two-electron oxidoreductases, but only one-electron reduction is considered responsible for the hypoxia selectivity of TPZ as this generates an oxygen-sensitive (reducing) radical that can eliminate H_2_O to generate powerfully oxidizing, cytotoxic benzotriazinyl (BTZ) or aryl radicals (Figure 1A) [15,24,25,26,27]. There are inconsistent reports in the literature as to where the critical TPZ one-electron reductases are located within the cell. Whilst TPZ is predominantly reduced by oxidoreductases that lie outside the nucleus (ca. 80% of total) [25], it has been postulated that multiple unidentified nuclear reductases are the mediators of TPZ-dependent DNA damage [28,29], suggesting that oxidoreductases located in other subcellular compartments do not play a constructive role in the activity of TPZ. Disagreement in the literature regarding the role of intra- versus extra-nuclear oxidoreductases in the hypoxic activation of TPZ and its analogues is exemplified by conflicting evidence around the role of NADPH:cytochrome P450 oxidoreductase (POR), an enzyme localized to the outer surface of the endoplasmic reticulum (ER) [30,31,32,33,34]. The ability of extra-nuclear reductases such as POR to elicit TPZ-induced DNA damage will depend on the diffusion range of TPZ-derived free radicals responsible for cytotoxicity.

Here we test the relevance of extra- versus intra-nuclear TPZ metabolism by expressing POR in different subcellular compartments. Relative sensitivities of parental MDA-231 cells and derivative cell lines engineered to overexpress POR in the nucleus, cytosol, endoplasmic reticulum or plasma membrane can shed light on the intracellular diffusion range of the TPZ radical and thus provide information on the fraction of TPZ radicals formed close enough to DNA to contribute to toxicity. We show that expression of POR in all subcellular compartments increases the sensitivity of the cells to TPZ under anoxic conditions, with only marginal increases in cytotoxicity observed when POR was expressed in the nucleus as opposed to other extra-nuclear cellular compartments. Unexpectedly, we also discover that increased nuclear metabolism, either through nuclear-localization of POR expression or nuclear-targeting of TPZ analogues [35], leads to a dramatic increase in aerobic cytotoxicity that negates hypoxia selectivity and which can be partially overcome by depletion of intracellular copper levels.

## 2. Results

### 2.1. Modelling Predicts That Formation of TPZ Radicals in Different Subcellular Compartments will Affect Efficiency of DNA Damage

To evaluate the likely intracellular diffusion range of the TPZ radical formed under hypoxia, we modelled its intracellular distribution following activation in different subcellular compartments assuming a radially symmetrical cell with radius 6.6 µm (Figure 1B). The reaction-diffusion model used the first order rate constants for elimination of H_2_O from the initial radical (*k_elim_*) and subsequent loss of the oxidizing radicals (*k_loss_*) as measured by pulse radiolysis [14], and radical disproportionation was neglected. Assuming an intracellular diffusion coefficient, *D*, of 4 × 10^−7^ cm^2^·s^−1^, corresponding to the measured average diffusion coefficient through HT29 multicellular layers [21], the distribution of oxidizing radicals is distinctly non-isotropic (Figure 1C); if activated in the ER, steady state concentrations in the nucleus are predicted to be 20–70% of the maximum, while if activated in at the inner surface of the plasma membrane the radicals are predicted to not reach the nucleus (Figure 1C). Given that *kl_oss_* >> *k_elim_* the normalized steady state distribution of the initial TPZ radical is essentially the same, and acts as the carrier for the cytotoxic oxidizing radicals. Given that intracellular *D* (assumed the same for TPZ and its radicals) is not well-defined, in Figure 1D we modelled higher and lower *D* values to reflect the wide range of reported values for cytoplasmic microviscosity [36,37]. This demonstrated that for PM activation *D* > 10^−6^ cm^2^·s^−1^ would be required to achieve nuclear radical concentrations >10% of that at the cell surface.

### 2.2. Generation of Cell Lines Expressing POR in Different Subcellular Compartments

**Figure 2:**
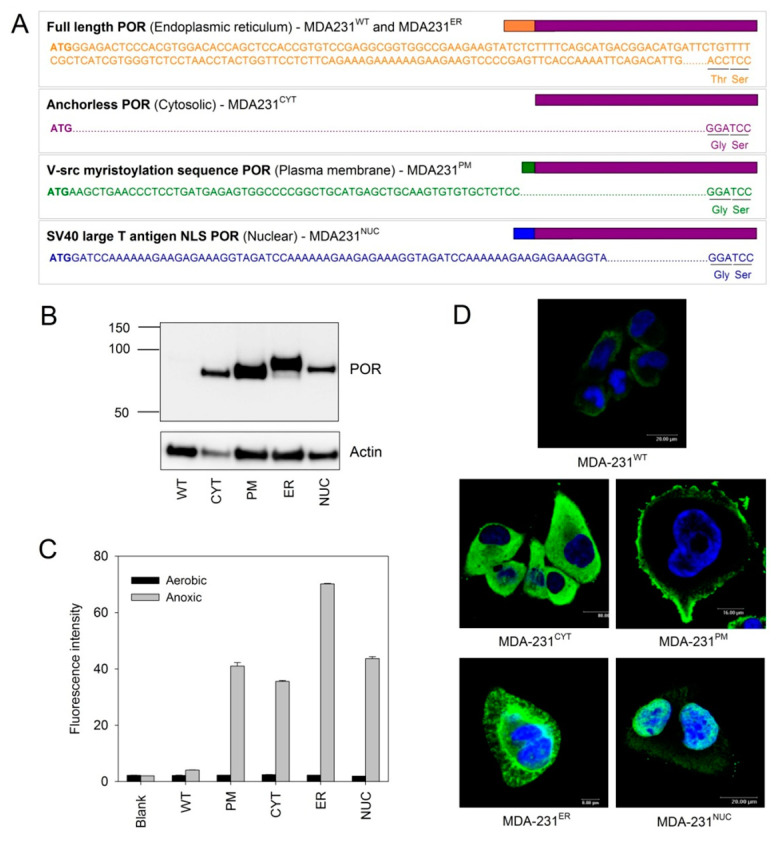
Generation of clonal MDA-231 cell lines expressing POR in different subcellular locations: (**A**) Details of the localization sequences used to direct POR to different subcellular compartments; (**B**) Western blot analysis showing expression levels and size differences of POR in the transfected cell lines; (**C**) Flow cytometry detection of POR enzyme activity in the transfected cell lines with an oxygen-sensitive fluorogenic POR substrate (FSL61). Results are the median fluorescence intensity of triplicate samples ± SEM. Blank is cell-free control; (**D**) Immunofluorescence microscopy showing the location of POR expression (green) in the MDA-231 cell lines. Nuclei are stained blue (Hoechst 33258).

In order to examine experimentally the extent that non-nuclear TPZ metabolism can contribute to DNA damage and cytotoxicity, MDA-231 clonal cell lines were constructed in which POR was expressed in four different subcellular compartments. To derive these cell lines, wild type MDA-231 (MDA-231^WT^) cells were transfected with POR-expressing plasmids in which the N-terminal ER anchor was either deleted (for cytosolic POR expression; MDA-231^CYT^) or replaced with alternate subcellular localization signals. Addition of the v-src localization signal (a myristoylation signal sequence) [38] enabled redirection to the inner plasma membrane (MDA-231^PM^) and addition of the SV40 large T antigen nuclear localization sequence enabled redirection to the nucleus (MDA-231^NUC^) [39]. A reference cell line was generated using a plasmid encoding wild-type POR (MDA-231^ER^) which localizes to the ER. The sequences of the modified localization signals are shown in Figure 2A.

Western blot analysis was performed to determine the relative expression of POR in each MDA-231 transfectant and to confirm that the size of each protein corresponded to the expected size based on the lengths of the N-terminal localization sequences used (Figure 2B). Functional POR activity was confirmed using the POR-selective fluorogenic probe, FSL61 [40]. Under anoxic conditions, MDA-231^WT^ cells showed only a minor increase in fluorescence when compared to cell-free (blank) control wells whilst POR over-expressing cell lines showed large increases in fluorescence (Figure 2C). No increase in FSL61 metabolism was observed under aerobic conditions. Immunocytochemical detection of POR by confocal microscopy confirmed POR was largely localized to the desired subcellular compartments (Figure 2D), with 3D imaging of cells confirming the majority of POR staining was localized to targeted regions (See Supplementary Movie Files).

### 2.3. Determination of TPZ Activation in MDA-231^WT^ and POR-Expressing Cells Variants

**Figure 3:**
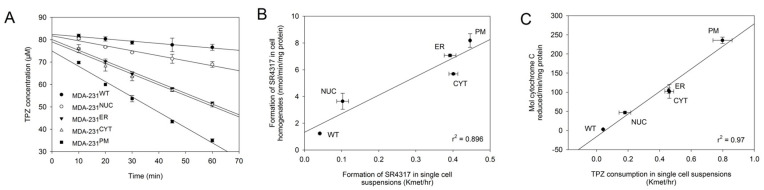
Expression of POR in different subcellular compartments results in increased TPZ activation: (**A**) Consumption of TPZ under anoxic conditions in parental and transfected MDA-231 cells. Results are the average of duplicate experiments, error bars indicate the range; (**B**) Correlation between the rates of anoxic formation of the non-toxic mono-*N*-oxide metabolite SR4317 in single cell suspensions (mean of duplicate samples) versus whole cell homogenates (mean of duplicate experiments). Error bars indicate the range in data; (**C**) Correlation between POR activity (rate of cytochrome C reduction in whole cell lysates; mean of duplicate experiments) and the rate of anoxic TPZ consumption in single cell suspensions (mean of duplicate experiments).

Parental and transfected cells were assessed for their ability to metabolize TPZ under anoxic conditions (Figure 3A). A concentration of 100 µM TPZ was selected to ensure first order non-saturable metabolism, as determined in previous studies [41]. Rates of TPZ consumption in single cell suspensions had rate constants (*k_met_*) of 0.042 h^−1^ (MDA-231^WT^), 0.180 h^−1^ (MDA-231^NUC^), 0.457 h^−1^ (MDA-231^ER^), 0.461 h^−1^ (MDA-231^CYT^) and 0.796 h^−1^ (MDA-231^PM^); the greatest rates were therefore observed in cells expressing POR at the plasma membrane and lowest rates observed in cells expressing POR in the nucleus. The differences in rates in MDA-231^PM^ and MDA-231^NUC^ cells could reflect differences in POR catalytic activity or they could reflect limited diffusion of TPZ into the nucleus. To distinguish between these possibilities, rates of anoxic formation of the mono-*N*-oxide metabolite SR4317 (a non-toxic two-electron reduction product) in single cell suspensions were compared with SR4317 metabolite formation in whole cell homogenates (Figure 3B). The correlation (r^2^ = 0.896) suggests that differences in rates in intact cells are due to differences in the levels of POR expression and not a result of prodrug gradients. To confirm this, the activity of POR was measured using the cytochrome C reduction assay and plotted against the rate of TPZ consumption in single cell suspensions: A strong correlation (r^2^ = 0.97) was observed between these parameters (Figure 3C).

### 2.4. One Electron Reduction of TPZ under Anoxia Yields DNA Damage Irrespective of the Subcellular Site of Activation

**Figure 4:**
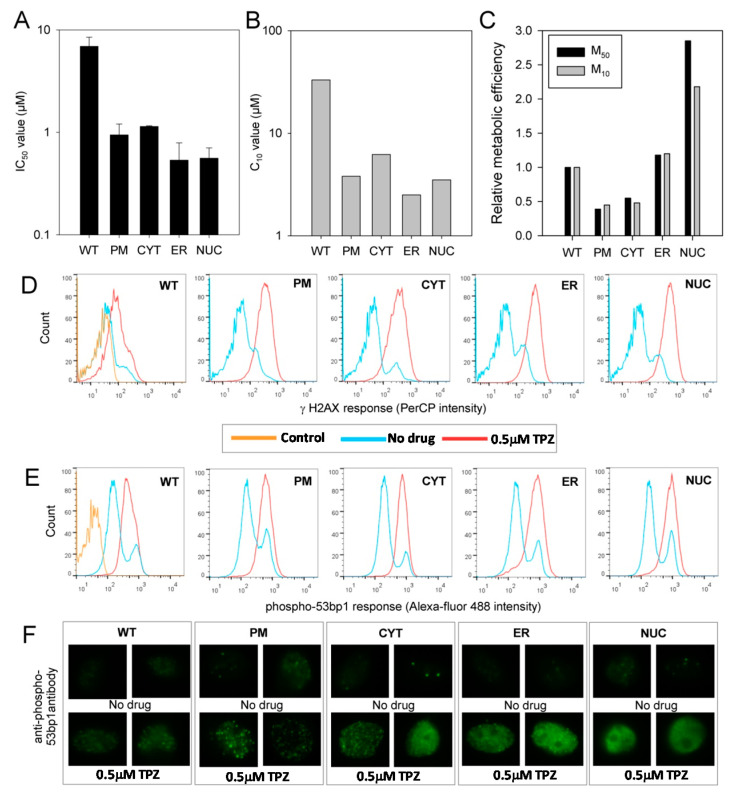
TPZ can achieve DNA damage under anoxia irrespective of the subcellular compartment of activation: (**A**) Sensitivity of parental and transfected MDA-231 cell lines to TPZ under anoxic conditions as determined by antiproliferative assay. IC_50_ values show the mean of 2–3 independent experiments, error bars show the SEM; (**B**) Sensitivity of parental and transfected MDA-231 cell lines to TPZ under anoxic conditions as determined by clonogenic assay. The concentration of TPZ required to reduce clonogenic survival by 90% (C_10_ values) were calculated in triplicate per drug concentration; (**C**) Relative metabolic efficiency in MDA-231 transfectants (extent to which metabolism contributes to cytotoxicity). M_50_ = 1/(IC_50_ × *k_met_*) of anoxic TPZ metabolism as derived from Figure 2A. M_10_ = 1/(C_10_ × *k_met_*) of anoxic TPZ metabolism as derived from Figure 2A. The M_50_ and M_10_ values were normalized to the value for MDA-231^WT^ cells; (**D**) Flow cytometry analysis of γ-H2AX levels in parental and transfected MDA-231 cells following a 4 h anoxic exposure to 0.5 µM TPZ and a 1 h drug-free recovery period under aerobic conditions. Control: no antibody labelling; (**E**) Flow cytometry analysis of phospho-53bp1 levels in parental and transfected MDA-231 cells following a 4 h anoxic exposure to 0.5 µM TPZ and a 1 h drug-free recovery period under aerobic conditions. Control: secondary antibody only; (**F**) Immunofluoresence microscopy showing phospho-53bp1 foci formation following a four-hour anoxic exposure to 0.5 µM TPZ and a 1 h drug-free recovery period under aerobic conditions.

The cytotoxicity of TPZ against the MDA-231 cell lines under anoxic conditions was examined using growth inhibition and clonogenic assay endpoints. Overexpression of POR in all subcellular compartments was shown to increase the sensitivity of the cells to anoxic TPZ exposure (Figure 4). The transfected cell lines showed growth inhibition IC_50_ values that were 6 to 12-fold lower than MDA-231^WT^ cells (Figure 4A); similar increases in sensitivity (9 to 13-fold) were seen in the C_10_ values of the clonogenic assay (Figure 4B). The endpoints of these assays were compared with the rates of anoxic TPZ consumption derived from Figure 3A to calculate the change in cellular sensitivity per unit of drug metabolism, i.e., metabolic efficiency (hereafter the term ‘metabolic efficiency’ will describe the relationship between cytotoxicity and metabolic consumption of TPZ). The relative metabolic efficiency for the cell lines was comparable when assessed using either the IC_50_ or the clonogenic assay endpoints (M_50_ and M_10_, respectively; Figure 4C). The metabolic efficiency in the MDA-231^ER^ cell lines was similar to that seen in MDA-231^WT^ cells; an expected result given POR is normally expressed in the endoplasmic reticulum. Expression of POR in the plasma membrane (MDA-231^PM^) or the cytosol (MDA-231^CYT^) caused the efficiency of anoxic cell kill to drop to about half the MDA-231^WT^ value. In contrast, the metabolic efficiency of MDA-231^NUC^ was 2 to 3-fold higher than in MDA-231^WT^ (or MDA-231^ER^) cells. Whilst TPZ reduction in the nuclei exhibits the greatest metabolic efficiency, reduction in non-nuclear compartments clearly contributes to anoxic cytotoxicity. However, the differences in metabolic efficiency (Figure 4C) suggest that the intracellular distribution of the TPZ radical is not strictly isotropic.

To confirm that the increased anoxic sensitivity observed in the POR expressing cell lines was due to DNA damage we assessed levels of γ-H2AX, a marker of unrepaired DNA double strand breaks, in a flow cytometry assay. A 4 h anoxic exposure to 0.5 µM TPZ increased levels of γ-H2AX by 2.5-fold in MDA-231^WT^ cells as compared to drug free controls. Larger increases in γ-H2AX levels following TPZ exposure were observed in the transfected cell lines: Whilst the greatest response was observed in MDA-231^NUC^ cells (8.4-fold), this was only marginally greater than that observed in MDA-231^ER^, MDA-231^CYT^ and MDA-231^PM^ cells (7.1, 5.1 and 5.4-fold, respectively) (Figure 4D). Flow cytometry was also used to examine levels of phospho-53bp1, a DNA damage marker that acts downstream of γ-H2AX. A four hour anoxic exposure to 0.5 µM TPZ generated similar results to that seen for γ-H2AX although the magnitude of the response was less. Levels of phospho-53bp1 versus drug-free controls were increased by 1.8, 2.4, 2.5, 2.2 and 2.3-fold in MDA-231^WT,^ MDA-231^ER^, MDA-231^CYT^ and MDA-231^PM^ and MDA-231^NUC^ cells, respectively (Figure 4E). Immunofluorescence microscopy was also used to detect distinct phospho-53bp1 foci in drug-free cells and cells exposed to 0.5 µM TPZ for 4 h (Figure 4F), the results of which confirmed observations from the flow cytometry analysis (Figure 4E).

### 2.5. TPZ Cytotoxicity under Aerobic Conditions Demonstrates Extreme Bias for Intranuclear Activation

**Figure 5:**
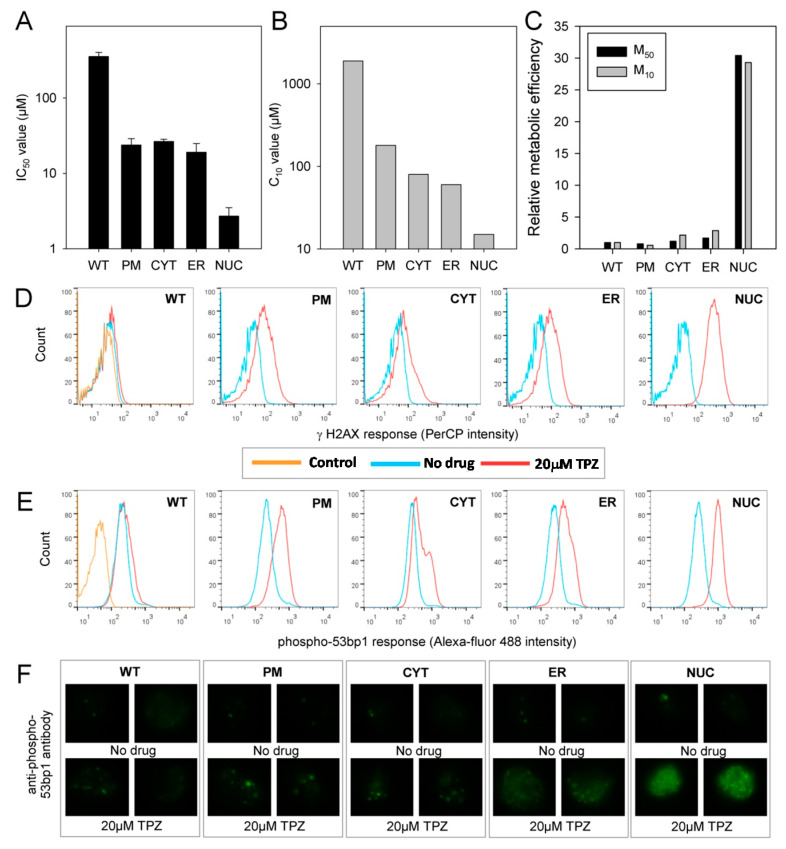
TPZ cytotoxicity under aerobic conditions displays major bias towards cells with POR localized in the nuclear compartment: (**A**) Sensitivity of parental and transfected MDA-231 cell lines to TPZ under aerobic conditions as determined by antiproliferative assay. IC_50_ values show the mean of 2-4 independent experiments, error bars show the SEM; (**B**) Sensitivity of parental and transfected MDA-231 cell lines to TPZ under aerobic conditions as determined by clonogenic assay. C_10_ values are derived from a single experiment (triplicate plates per drug concentration); (**C**) Relative metabolic efficiency in MDA-231 transfectants (extent to which productive metabolism contributes to cytotoxicity). M_50_ = 1/(IC_50_ × *k_met_*) of anoxic TPZ activity as derived from Figure 3A. M_10_ = 1/(C_10_ × *k_met_*) of anoxic TPZ activity as derived from Figure 3A). The M_50_ and C_10_ values were normalised to the value for MDA-231^WT^ cells; (**D**) Flow cytometry analysis of γ-H2AX levels in parental and transfected MDA-231 cells following a 4 h aerobic exposure to 20 µM TPZ and a 1 h drug-free recovery period under aerobic conditions. Control is no antibody labelling; (**E**) Flow cytometry analysis of phospho-53bp1 levels in parental and transfected MDA-231 cells following a 4 h aerobic exposure to 20 µM TPZ and a 1 h drug-free recovery period under aerobic conditions. Control is secondary antibody only; (**F**) Immunofluoresence microscopy showing phospho-53bp1 foci formation following a four hour aerobic exposure to 20 µM TPZ and a 1 h drug-free recovery period under aerobic conditions.

The potential for TPZ to inhibit growth of cells under aerobic conditions was assessed by IC_50_ assay and clonogenic assay (Figure 5).

Expressing POR in the endoplasmic reticulum, cytosol or plasma membrane increased aerobic sensitivity to TPZ in the range of 11 to 32-fold with a trend favoring proximity to the nucleus (ER > CYT > PM). In contrast to anoxic activation, a larger increase in aerobic sensitivity to TPZ of ~130-fold was observed when POR was expressed in the nucleus (Figure 5A,B). As a direct consequence, the hypoxic cytotoxicity ratio (HCR; ratio of anoxic sensitivity to aerobic sensitivity) collapsed from ~50-fold in MDA-231^WT^ cells to ~5-fold in MDA-231^NUC^ cells. When estimating the ‘metabolic efficiency’ of TPZ in the IC_50_ and clonogenic assays under aerobic conditions, it is necessary to estimate the total rate of TPZ reduction and subsequent superoxide formation, due to the absence of net TPZ consumption as a consequence of futile redox cycling. The rate of anoxic TPZ loss (derived from Figure 3A) is a suitable surrogate for estimating the rate of TPZ redox cycling. Using this approach, M_50_ and M_10_ values in MDA-231^NUC^ cells were 30-fold higher than those seen in MDA-231^WT^ cells, considerably greater than when POR is expressed in any other cellular compartment (Figure 5C).

We next assessed levels of γ-H2AX by flow cytometry. A 4 h exposure to 20 µM TPZ under aerobic conditions did not alter γ-H2AX levels in MDA-231^WT^ cells as compared to drug-free controls. Increases in γ-H2AX following aerobic TPZ exposure were observed in MDA-231^ER^, MDA-231^CYT^ and MDA-231^PM^ cells (3.6, 2.6 and 3.8-fold, respectively), however, these increases were considerably lower than the 16.1-fold increase in γ-H2AX observed in MDA-231^NUC^ cells (Figure 5D). Flow cytometry analysis of phospho-53bp1 levels showed results consistent with γ-H2AX although the magnitude of signal was less; no changes were observed in MDA-231^WT^ cells, small increases were observed in MDA-231^ER^, MDA-231^CYT^ and MDA-231^PM^ cells (2.1, 1.8 and 2.2-fold, respectively) and the largest change occurred in MDA-231^NUC^ cells (3.3-fold) (Figure 5E). Consistently, immunofluorescence analysis showed an increase in the number of phospho-53bp1 foci following TPZ treatment in the transfected cell lines, with the highest intensity observed in MDA-231^NUC^ cells (Figure 5F).

### 2.6. Copper May Play an Important Role in the Increased Aerobic TPZ Toxicity Observed in MDA-231^NUC^ Cells

**Figure 6:**
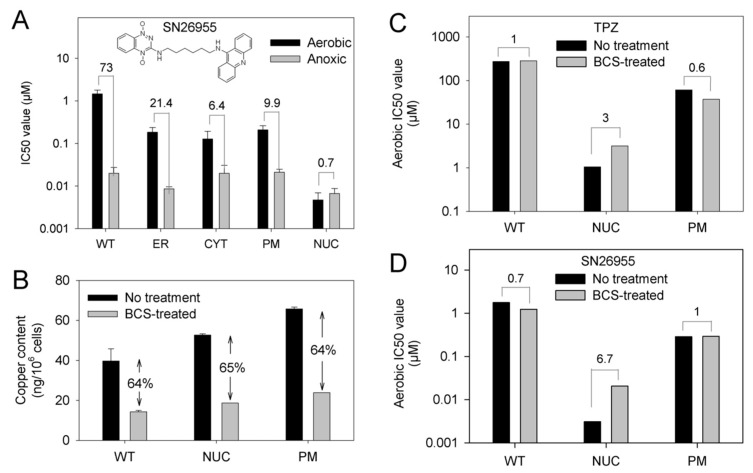
Aerobic toxicity of TPZ and an analogue with increased affinity for DNA is mediated by a copper-dependent mechanism in MDA-231^NUC^ cells: (**A**) Sensitivity of parental and transfected MDA-231 cell lines to SN26955, which contains a TPZ moiety linked to a DNA intercalator, under aerobic conditions as determined by antiproliferative assay. IC_50_ values show the mean of 3-4 independent experiments, error bars show the SEM; numbers are the HCR values. (**B**) Quantitation of the copper content of cells incubated for 7 days in normal media or media supplemented with the copper chelating agent bathocuproine (BCS) as determined by ICP-MS analysis. (**C**) Effects on chronic BCS exposure on the aerobic sensitivity of parental, MDA-231^NUC^ or MDA-231^PM^ cells to TPZ as determined by anti-proliferative assay. (**D**) Effects on chronic BCS exposure on the aerobic sensitivity of parental, MDA-231^NUC^ or MDA-231^PM^ cells to SN26955 as determined by anti-proliferative assay.

The observation that intranuclear reduction of TPZ enhances aerobic more than anoxic cytotoxicity led us to examine whether attachment of a DNA-affinic acridine chromophore to the TPZ moiety, could exaggerate aerobic response in the MDA-231^NUC^ cells (Figure 6A). Antiproliferative assays with the TPZ analogue SN26955 [35] showed the HCR collapsed to below unity in the MDA-231^NUC^ cells, with the drug showing greater toxicity under aerobic conditions than under anoxic conditions (Figure 6A). In contrast, the MDA-231^WT^ cell line retained hypoxia selectivity, exhibiting an HCR of 73. Expression of POR in other cellular compartments increased sensitivity of the cells to SN26955; hypoxic selectivity was retained (Figure 6A).

We hypothesized that the presence of metal ions may contribute to this toxicity. To examine this possibility we used the cell-excluded copper chelator bathocuproine (BCS) to deplete copper in MDA-231 cells prior to TPZ treatment. Following chronic exposure to BCS for 7 days (100 µM for 4 days followed by 300 µM for 3 days) total cellular copper content was measured by ICP-MS analysis with reference to a copper standard curve. MDA-231^WT^, MDA-231^NUC^ and MDA-231^PM^ cells all showed a similar response to BCS exposure with a ~64% decrease in copper content (Figure 6B). Cells were next exposed to 300 µM BCS for 7 days prior to determining their sensitivity to TPZ (Figure 6C) or SN26955 (Figure 6D) under aerobic conditions by antiproliferative assay. No change in aerobic sensitivity was observed in MDA-231^WT^ or MDA-231^PM^ cells to either drug following BCS exposure. In contrast, MDA-231^NUC^ cells were 3-fold less sensitive to TPZ (Figure 6C) and 6.7-fold less sensitive to SN26955 (Figure 6D) after BCS treatment.

## 3. Discussion

Understanding the enzymology of hypoxia-activated prodrugs can help target treatment to appropriate patient groups and assist in rational development of next generation agents [42]. The sub-cellular location of drug-activating enzymes can play an important role in drug sensitivity. For example, expression of mitochondrial NADH: cytochrome *b*_5_ reductase (CYB5R) in different sub-cellular compartments alters sensitivity of cells to mitomycin C (MMC) [43,44]. Overexpression of a cytosolic CYB5R restored sensitivity to MMC in air and increased hypoxic drug sensitivity [43]. Furthermore, targeting CYB5R expression to the nuclear compartment resulted in increased drug sensitivity under both aerobic and hypoxic conditions [44].

Here we have generated isogenic MDA-231 cell lines to provide information on the relevance of TPZ activation and subsequent radical diffusion in different subcellular compartments. MDA-231 cells exhibit a low endogenous POR activity [45] and thus provide a suitable background for ectopic POR expression. Confocal microscopy and use of a fluorogenic POR probe was used to confirm the subcellular location of POR expression and functional POR activity, respectively (Figure 2 and Supplementary Movie Files). Immunostaining outside the targeted regions is likely to reflect active protein trafficking of POR. Increased anoxic TPZ consumption was observed in all the transfected cells as compared to MDA-231^WT^ cells, with the highest rates observed in MDA-231^PM^ cells and the lowest rates observed in MDA-231^NUC^ cells (Figure 3A). Because of the low lipophilicity of TPZ (logP = −0.32) [46], the apparent differences in TPZ consumption could, in principal, be affected by prodrug gradients caused by cellular membranes acting as barriers to TPZ diffusion. However, the differences in TPZ consumption were confirmed to be a direct result of differences in enzyme expression (Figure 3B,C). In contrast to previous reports [29], constructive metabolism of TPZ appears not to be restricted to the nucleus under anoxic conditions, although TPZ reduction in non-nuclear compartments resulted in a lower cytotoxic efficiency than TPZ reduction in the nucleus (Figure 4). Analysis of DNA damage response markers (γH2AX and phospho-53bp1; Figure 4) indicate that TPZ-mediated cell death under anoxic conditions was consistently due to induction of DNA double strand breaks irrespective of the subcellular site of TPZ reduction. Therefore, the TPZ radical, as a carrier for the ultimate oxidizing radicals, appears capable of diffusing significant distances across the cell under anoxic conditions. However, the apparent mismatch between experimental estimates of metabolic efficiency (Figure 4C) and theoretical calculations showing limited radical diffusion (Figure 1C) suggests that the experimental values of M10 may overestimate diffusion range because of incomplete targeting of POR to intended sub-cellular compartments.

Little is known about the mechanisms of aerobic TPZ toxicity, though it is likely to account for many of the significant clinical side effects of TPZ [47,48,49,50]. In the present study, an increase in nuclear TPZ reduction caused a much larger increase in relative metabolic efficiency under aerobic conditions (~30-fold; Figure 5C) than under anoxia (~2–3-fold, Figure 4C) and an associated collapse in the HCR from ca. 50-fold in MDA-231^WT^ cells to less than 5-fold in MDA-231^NUC^ cells. This finding is highly unexpected and contradicts the proposal that intra-nuclear metabolism of TPZ is the major mechanism of hypoxia selectivity [29]. Further, exposing MDA-231^NUC^ cells to the TPZ analogue SN26955 [35], a chemical-biology tool with high affinity for intercalative binding to DNA, strongly exacerbates this phenomenon. Consequently toxicity was greater following aerobic exposure (HCR = 0.7) thus collapsing hypoxic-selectivity entirely. These results indicate that extreme cytotoxicity manifests when single-electron donation to TPZ or SN26955, followed by back oxidation, occurs in a setting that is intimately associated with DNA.

While futile metabolic reduction and back-oxidation of TPZ results in no net consumption of substrate, it fuels formation of superoxide radicals that are readily converted to H_2_O_2_ by superoxide dismutase. Copper ions can play a key role in the conversion of H_2_O_2_ to short lived DNA-damaging hydroxyl radicals in Fenton-like chemical reactions [51] and the generation of very reactive and short-lived hydroxyl radicals by Cu(II) is implicated in DNA damage by H_2_O_2_ [52]. Numerous studies have shown that transition metals can facilitate the generation of the hydroxyl radical leading to oxidative DNA damage [53,54,55]. We speculated that copper ions are likely to play a key role in H_2_O_2-_mediated DNA damage in the context of aerobic TPZ toxicity. The nucleus holds ~20% of the copper contained within the cell [56], which plays an integral role in preserving the structure of the nuclear matrix [57,58], binds electrostatically to DNA [56,59] and stabilizes complexes between DNA and topoisomerase II and other matrix proteins [60,61,62]. Depletion of copper using BCS, a cell excluded Cu(I) chelator [63], protected MDA-231^NUC^ cells, but not MDA-231^WT^ or MDA-231^PM^ cells, from aerobic exposure to TPZ or SN26955 (Figure 6C,D). The data presented here highlight the importance of cellular copper levels in aerobic TPZ toxicity when reduction occurs predominantly in the nucleus. This supports the notion that the aerobic toxicity of TPZ is due, in part, to a nuclear-localized transition metal dependent redox phenomenon.

These data establish, for the first time, that anoxic TPZ cytotoxicity is much less constrained by nuclear metabolism than is oxic cytotoxicity. Rather, nuclear activation of TPZ in the presence of oxygen has a dramatic cytotoxic effect, an observation that has important implications for the mechanisms of toxicity of this class of agent. This novel work casts doubt on the veracity of previous findings that implicate nuclear reductases as the exclusive source of biologically relevant hypoxic metabolism [28,29] and suggests that attempts to develop TPZ analogues with increased nuclear activation [35] would be detrimental to the hypoxic-specificity of the drug. In support, recent genome-wide screening found that POR is the major determinant of sensitivity for SN30000 [34] indicating that metabolism of this clinical stage TPZ analogue is not driven by intra-nuclear oxidoreductases. Elucidation of the precise enzymology of TPZ could result in the identification of additional analogs which are not activated by nuclear enzymes and this may improve hypoxia-selectivity, perhaps leading to fewer side effects in the clinic.

The findings from this current study also highlight a potential clinical development path for TPZ, SN30000 or related analogues in combination with copper chelating agents such as trientine or tetrathiomolybdate. Copper chelating agents are commonly used for the treatment of Wilson’s disease, which is caused by a genetic defect that impairs copper metabolism [64]. However, elevated copper levels have also been observed in cancer patients [65], and copper chelation has been proposed as an anti-angiogenic strategy for cancer treatment [66]. Both trientine [67,68] and tetrathiomolybdate [69,70] are demonstrated to reduce angiogenesis and suppress tumor development. Phase II clinical trial of tetrathiomolybdate was conducted in patients with breast cancer, for which it was hypothesized that copper depletion would result in a reduction of VEGFR2-positive endothelial progenitor cells, alterations to the tumor microenvironment and improvements in event-free and overall survival [71]. This study concluded that tetrathiomolybdate could be safely administered over a two-year period, was well tolerated and affected copper-dependent components of the tumor microenvironment. Thus, there is a clear rationale for combining copper chelating agents with TPZ, SN30000 or related analogues in a clinical setting. Based on the data presented here, copper depletion would be expected to reduce cytotoxicity under aerobic conditions whilst having minimal consequences under hypoxic conditions. Copper depletion would therefore likely reduce the normal tissue toxicities associated with this class of HAP and lead to improved tolerance with the potential for dose escalation. The anti-angiogenic effect of copper chelators may also reduce oxygen supply to the tumor, increasing the proportion of hypoxic tumor cells that would be targets for TPZ or SN30000, and thereby offering the potential for synergy between these agents.

## 4. Materials and Methods

### 4.1. Compounds

Tirapazamine (TPZ), SN26955 [35,72] and FSL61 [73] were synthesized in the Auckland Cancer Society Research Centre as reported previously. Stock solutions were prepared in DMSO and stored at −20 °C. Stock solutions of bathocuproine disulphonic acid (BCS, Sigma Aldrich, St. Louis, Mo, USA) were prepared in 1× PBS, filter sterilized, and stored at 4 °C in darkness before dilution to the desired concentration in α-minimal essential medium (α-MEM) immediately before use.

### 4.2. Intracellular Diffusion of TPZ and Its Radicals

Intracellular reaction-diffusion of TPZ and its radicals were simulated using a simplified reaction scheme (TPZ → TPZ radical → oxidizing radicals → inactive products; See Figure 1A) in a 1.2 pL spherical cell (radius 6.6 µm). The reaction-diffusion equations with respect to time, *t*, and radial distance, *r*, for this scheme are:

(1) $$\frac{\partial TPZ}{\partial t}=D\frac{\partial^{2}TPZ}{\partial x^{2}}-\frac{2}{r_{max}-r}D\frac{\partial TPZ}{\partial t}-k_{met}TPZ$$

(2) $$\frac{\partial TPZ_{r}}{\partial t}=D\frac{\partial^{2}TPZ_{r}}{\partial x^{2}}-\frac{2}{r_{max}-r}D\frac{\partial TPZ_{r}}{\partial t}+k_{met}TPZ-k_{elim}TPZ_{r}$$

(3) $$\frac{\partial TPZ_{o}}{\partial t}=D\frac{\partial^{2}TPZ_{o}}{\partial x^{2}}-\frac{2}{r_{max}-r}D\frac{\partial TPZ_{o}}{\partial t}+k_{elim}TPZ_{r}-k_{loss}TPZ_{o}\;\;\;\;\;\;\;\;\;\;\;\;\;\;\;\;\;\;\;\;$$

where *TPZ_r_* is the concentration of the initial (reducing) TPZ radical, *TPZ_o_* represents the concentration of the oxidising radicals, *r_max_* is the radius of the cell, and *D* is the intracellular diffusion coefficient of TPZ and its downstream radicals. Boundary conditions were no flux at the plasma membrane and centre of the cell. TPZ concentration was fixed at 100 µM at the cell surface and initial conditions were *TPZ_r_* = *TPZ_o_* = 0 µM throughout the cell. The system of partial differential equations was solved numerically using an adaptive finite difference method in the function NDsolve supplied with Mathematica^TM^ v11.1 (Wolfram, Champaign, IL, USA), with TPZ and radical concentrations plotted at 60s, after steady state had been achieved.

TPZ enzymatic bioreductive metabolism under anoxia was assumed to be localised to particular regions (Figure 1C) to represent the ER (MDA-231^WT^ and MDA-231^ER^ cells), a region 1 µm inside the plasma membrane (MDA-231^PM^ cells), the nucleus or the cytoplasm. The rate constant for this TPZ metabolism to the initial radical (*k_met_*) was set to the values measured in each cell line (Table 1), scaled to the volume of the appropriate compartment, assuming a cell volume fraction in stirred cell suspensions at 5 × 10^5^ cells/mL of 0.6 pL/mL. The rate constant for conversion of the TPZ radical to the DNA damaging radical (*k_elim_*) was set to 100 s^−1^ and the rate constant for loss of the damaging radical (*k_loss_*) was set to 10^4^ s^−1^ consistent with rates determined by pulse radiolysis [14,15]. All radical reactions were assumed to occur homogeneously throughout the cell and radicals were assumed to be permeable to the nucleus but impermeable to the plasma membrane. The diffusion coefficient of all species in cytoplasm was set initially to 4 × 10^−7^ cm^2^·s^−1^ based on the diffusion coefficient of TPZ in HT29 multicellular layers [74]. Given that this value may be influenced by paracellular transport and by plasma membrane barriers, its applicability is uncertain so further simulations were performed using a range of values of *D*.

### 4.3. Cell Lines

Cells were maintained in culture under humidified atmospheric conditions with 21% O_2_ and 5% CO_2_ [75,76] as described with < 3 month cumulative passage from authenticated stocks. MDA-MB-231 cells were sourced from Manchester University (UK) and STR phenotyped to confirm authenticity (CellBank, Sydney, Australia). Harvested cells were counted with an electronic particle counter (Z2 Coulter Particle Analyzer, Beckman Coulter, Miami, FL, USA). Sequences encoding POR, either the full length gene or with modified localisation sequences, were cloned into F527, a modified version of pEF-IRES [77]. Transfected cells were selected using puromycin and individual clonal cell lines generated.

### 4.4. Western Immunoblot Analysis

Cell lysates were prepared in radioimmunoprecipitation assay buffer. 30 µg of protein was loaded on SDS-polyacrylamide electrophoresis gels (4–12% gradient gels; Invitrogen, Carlsbad, CA, USA), transferred, blocked, and probed with primary antibodies against POR (Santa Cruz Biotechnology, Inc., Santa Cruz, CA, USA). Bands were detected using chemiluminescent ECL detection (SuperSignal; Thermo Fisher Scientific, Waltham, MA, USA) and quantified using ImageJ (version 1.42 of the public domain software).

### 4.5. Characterization of POR Expressing Cells by Immunofluorescence Microscopy

Cells were grown on polylysine coated coverslips to 50% confluency. Media was removed by PBS wash (3×) and cells fixed with 100% methanol. Cells were washed 3× with PBS containing 1% normal goat serum (NGS). Cells were incubated in PBS containing 1% NGS and an anti-POR primary antibody (Daiichi pure chemicals, Tokyo, Japan) for 1 h at room temperature. After washing in PBS (4×), cells were incubated in PBS containing 1% NGS and the rabbit anti-goat secondary antibody (FITC conjugated) and incubated for 1 h at 4 °C. Cells were washed in PBS (3×) before being exposed to 0.5 µg/mL Hoechst 33258 (Sigma Aldrich, St. Louis, Mo, USA) in PBS for 15 min at room temperature to act as a nuclear counterstain. Coverslips were mounted to a glass slide using Vectashield mounting medium (Vector Laboratories, Birlingame, CA, USA). Images were captured using a TE2000E inverted microscope (Nikon). Sequential Z plane images were stacked to generate 3D images.

### 4.6. Characterization of POR-Expressing Cells Using the Fluorogenic Probe FSL61

Cells were analyzed by flow cytometry for POR activity using the fluorogenic probe FSL61 as described previously [40,73]. Cells were harvested, counted and resuspended to a density of 1 × 10^6^ cells/mL under aerobic or anoxic conditions. 500 μL of the appropriate cells were seeded in triplicate in wells of 24-well non-tissue culture treated plates and incubated at 37 °C for 1 h. 100 μL of media containing FSL61 was added (final concentration 300 μM) and cells were incubated for 3 h. The cell suspension was then transferred to mL tubes before analysis on a BD LSRII flow cytometer (BD Biosciences, San diego, CA, USA).

### 4.7. Drug Metabolism in Single Cell Suspensions

Cells were harvested, counted and resuspended in αMEM (minus FCS) to a density of 5 × 10^5^ cells/mL. 5–10 mL of each cell suspension were transferred to 25 mL glass bottles containing magnetic stir bars and placed in a 37 °C water bath with a gassing manifold. Cells were gassed for 1 h using humidified oxygenated (95% compressed air, 5% CO_2_) or anoxic (95% N_2_, 5% CO_2_) gas supplies. TPZ (final concentration 100 µM) was added and 0.3 mL samples were collected at 10, 20, 30, 45 and 60 min. Each sample was chilled on ice for 1 min then centrifuged (7600× *g* for 1 min). Supernatants were transferred to fresh eppendorf tubes and stored at −80 °C until HPLC analysis. An Agilent 1100 HPLC system with diode array detector (Agilent Technologies, Waldbronn, Germany) was used to measure concentrations of the drug and its two-electron reduced metabolite SR4317 in extracellular samples as described previously [21,41]. Samples were run alongside standard solutions of TPZ and SR4317.

### 4.8. Determination of Enzyme Activity

The activity of POR in the cell lines was measured in S-9 fractions prepared as described [75,78]. Total protein concentration was measured using the BCA assay [79]. POR activity was determined by spectrophotometric assay as the cyanide-resistant reduction of cytochrome c in the presence of NADPH, as described [75].

### 4.9. Antiproliferative (Cytotoxicity) Assays

Cells were seeded into 96-well plates (1500 cells/0.1 mL). Assays were performed in αMEM supplemented with 10% FCS, 200 µM 2′ deoxycytidine and 10 mM D-glucose under oxic or anoxic conditions, the latter using a 5% H_2_/palladium catalyst scrubbed Bactron anaerobic chamber (Sheldon Manufacturing, Cornelius, OR, USA) to achieve strict anoxia (<1 ppm O_2_ gas phase) during TPZ exposure as described previously [75]. Total exposure to anoxia did not exceed 6 h, and cells were allowed to regrow for 5 days under drug-free aerobic conditions. Cultures were then stained with sulphorhodamine B (SRB) to measure total cells [80]. The IC50 was determined by interpolation as the drug concentration reducing staining to 50% of controls on the same plate.

### 4.10. Clonogenic Assays

Cell killing was assessed by clonogenic assay (loss of colony forming potential). Cells were harvested, counted and resuspended in αMEM supplemented with 10% FCS, 200 µM 2′-deoxycytidine and 10 mM D-glucose to a density of 1 × 10^5^ cells/mL. 8 mL of cell suspension was transferred to glass bottles containing magnetic stir bars and placed in a 37 °C water bath with a gassing manifold. Cells were gassed for 1 h using humidified oxygenated (95% compressed air, 5% CO_2_) or anoxic (95% N_2_, 5% CO_2_) gas supplies. Following drug addition, cells were incubated under the same conditions for 1 h. Samples were chilled on ice, 7 mL transferred to fresh tubes and centrifuged at 45× *g* for 5 min. Cells were resuspended in drug-free media to a density of 100,000 cells/mL and a series of 10-fold dilutions prepared down to 100 cells/mL. 1 mL of each dilution was plated in triplicate into 60 mm cell culture dishes containing 5 mL culture media (αMEM with 5% FBS). The plates were incubated at 37 °C for 13 days, stained with methylene blue and colonies with > 50 cells were counted. Surviving fraction was calculated as the ratio of colonies from treated/control vials.

### 4.11. Detection of phospho-53bp1 or γH2AX by Flow Cytometry

Cells were seeded into 6-well plates under aerobic or anoxic conditions to a density of 1 × 10^6^ cells in 4 mL media. Cells were incubated for 2 h prior to addition of TPZ. Following a 4 h drug incubation the medium was replaced with drug-free medium and the samples incubated under aerobic conditions for 1 h. For γH2AX staining cells were fixed and processed as per the instructions in the FlowCellect DNA damage histone H2A.X dual detection kit (MilliporeSigma, St. Louis, Mo, USA). For 53bp1 staining, cells were detached with trypsin, washed twice with 3 mL cold PBS and fixed in 3 mL cold PBS/2% paraformaldehyde for 20 min. Cells were washed with 3 mL PBS/1% BSA and permeabilized in 3 mL PBS/1% BSA/0.2% triton X-100 for 20 min at room temperature. Cells were washed in 3 mL PBS/1% BSA and blocked in PBS/1% BSA/5% normal goat serum (NGS) for 30 min. Blocking buffer was removed and cells were resuspended in 300 µl PBS/1% BSA/1% NGS containing the phospho-53bp1 antibody (1:100 dilution; Cell signalling technologies) and incubated overnight at 4 °C. Cells were washed 3 times in 2 mL PBS/1% BSA then resuspended in 300 µl PBS/1% BSA/1% NGS containing a goat anti-rabbit alexafluor 488 antibody (1:200 dilution; Invitrogen) for 2 h at room temperature. The antibody was removed with 3 washes of 2 mL PBS prior to analysis. All samples were analysed on a BD LSRII flow cytometer (BD Biosciences).

### 4.12. Detection of phospho-53bp1 foci by Immunofluorescence Microscopy

Cells were seeded onto poly-lysine treated glass coverslips in 24 well plates and incubated overnight. Anoxic samples were transferred to the anaerobic chamber and equilibrated for 2 h. Cells were incubated for 4 h in the presence or absence of TPZ under aerobic and anoxic conditions. Following drug treatment, media was replaced with fresh drug-free media and the cells incubated for 1 h under aerobic conditions. After a 1 h incubation cells were washed in PBS (3×) and fixed in cold PBS/2% paraformaldehyde for 20 min at room temperature. Cells were washed with PBS/1% BSA (3×) and permeabilized in PBS/1% BSA/0.2% Triton-X100 for 10 min. After 3 washes with PBS/1% BSA a blocking buffer (PBS/1% BSA/5% normal goat serum) was applied for 30 min before addition of the primary antibody, rabbit phospho-53BP1 Ser1778 (Cell Signaling Technologies, Danvers, MA, USA) in PBS/1% BSA/1% normal goat serum. Cells were incubated in primary antibody at 4 °C overnight, washed in PBS/1% BSA (3×) and incubated with secondary antibody (goat anti-rabbit alexafluor 488; Invitrogen) for 2 h at room temperature. The nucleus was stained with DAPI (Invitrogen, Carlsbad, CA, USA) and cells washed 4 times with PBS prior to mounting on microscope slides using prolong gold anti-fade reagent (Invitrogen). Images were taken on a DMR microscope (Leica, Wetzlar, Germany).

### 4.13. Inductively Coupled Plasma–Mass Spectrometry (ICP-MS) of Intracellular Copper

Cells were harvested, washed twice in PBS and cell pellets frozen at −80 °C. Thawed pellets were resuspended in 70% *v*/*v* HNO_3_ (final concentration 1 × 10^6^ cells/mL). Samples were held overnight in a fume hood at room temperature. On the following day, cell lysates were further digested for 2 h at 70 °C (Thermomixer, Eppendorf, Hamburg, Germany). Immediately prior to analysis, lysates were diluted 4-fold in polyethylene tubes using platinum-spiked Milli-Q water (1.5 ppb Pt) to reach a final concentration of 2.5 × 10^5^ cells/mL in samples introduced to the inductively coupled plasma mass spectrometer (ICP-MS). Polyethylene tubes were thoroughly cleaned by soaking in 1% *v*/*v* nitric acid for at least 24 h followed by rinsing in Milli-Q water and allowed to dry completely before use. Blanks, copper standards and biological samples were read after tuning the ICP-MS to copper. Quality control samples (containing 1 ppb Cu) were scattered amidst samples to monitor for any potential instrument drift. The ICP-MS system employed was a Hewlett Packard ^®^ HP4500 series (Agilent Technologies, Yokohama, Japan) with a Babington (v-groove) nebuliser and a Scott double-pass spray chamber maintained at 2 °C. The operating conditions were as previously described with minor modifications. Briefly, four mass ranges were analysed, accommodating for Cu^63^, Cu^65^, Pt^194^ and Pt^195^ respectively. By comparing the counts obtained for each isotope against their known biological abundance (Cu^63^ = 69.2%; Cu^65^ = 30.8%), the continuous monitoring of total counts obtained was enabled. The peristaltic pump uptake speed and sample uptake speeds were both set at 0.1 mL/min, and the rinse duration between samples was 30 s. Copper tuning solution (10 ppb Cu in 1% *v*/*v* HNO_3,_ Riedel de Haen, AG, Seelze, Germany) and copper standards (known concentrations in 70% *v*/*v* HNO_3_) were prepared from CuSO_4_. Copper standards were diluted appropriately in Milli-Q water at the same time as digested biological samples to avoid matrix effects.

## 5. Conclusions

These data question the previous finding that nuclear metabolism of tirapazamine is responsible for the complex DNA damage associated with hypoxia-selective cytotoxicity. Rather, elevation of one-electron reductase activity within the cellular nucleus has the opposing effect, rendering cell hypersensitive to the aerobic effects of TPZ exposure. This body of work emphasizes that all cellular compartments are relevant for the bioactivation of TPZ and sheds new light on the design and development of next generation analogues.

## Figures and Tables

**Table 1:** Rate constants for TPZ metabolism in POR-expressing MDA-231 cells under anoxia.

	MDA-231^POR^	MDA-231^ER^	MDA-231^NUC^	MDA-231^CYT^	MDA-231^WT^
*k_met_* ^a^	0.947 s^−1^	0.816 s^−1^	2.25 s^−1^	0.222 s^−1^	0.075 s^−1^
Compartment dimensions ^b^	0 < *r* < 1 µm	2.2 < *r* < 4.4 µm	4.4 < *r* < 6.6 µm	0 < *r* < 4.4 µm	2.2 < *r* < 4.4 µm
Compartmentvolume fraction	0.389	0.259	0.037	0.963	0.259

^a^ Values from Figure 3A scaled to the volume of the appropriate compartment. ^b^ Radial inward distance.

## Supplementary Materials

The following are available online. Video S1: Single cell immunohistochemistry of POR expression in MDA-231^PM^ and MDA-231^NUC^ using a TE2000E inverted microscope with images sequential Z plane stacked to generate 3D images.

## Abbreviations

αMEM:	Alpha-minimal essential media
BCA:	Bicinchoninic acid
BCS:	Bathocuproine disulphonic acid
BTZ:	Benzotriazinyl
CYB5R:	NADH Cytochrome *b*_5_ reductase
CYT:	Cytosol
DAPI	4′,6-Diamidino-2-phenylindole
ER:	Endoplasmic reticulum
FMISO:	Fluoromisonidazole
HAP:	Hypoxia-activated prodrug
HCR:	Hypoxic cytotoxicity ratio
HNSCC:	Head and neck squamous cell carcinoma
HPLC:	High performance liquid chromatography
HPV:	Human papilloma virus
ICP-MS:	Inductively coupled plasma-mass spectrometry
MMC:	Mitomycin C
NGS:	Normal goat serum
NSCLC:	Non-small cell lung cancer
NUC:	Nuclear
PET:	Positron Emission Tomography
PM:	Plasma membrane
POR:	NADPH:Cytochrome P450 oxidoreductase
SRB:	Sulphorhodamine B
TPZ:	Tirapazamine
